# Mediterranean dietary pattern and VEGF +405 G/C gene polymorphisms in patients with metabolic syndrome: An aspect of gene-nutrient interaction

**DOI:** 10.1371/journal.pone.0171637

**Published:** 2017-02-17

**Authors:** Ghazaleh Hajiluian, Mahdieh Abbasalizad Farhangi, Leila Jahangiry

**Affiliations:** 1 Student Research Committee, Nutrition Research Center, Faculty of Nutrition, Tabriz University of Medical Sciences, Tabriz, Iran; 2 Nutrition Research Center, Drug Applied Research Center, Department of Community Nutrition, Tabriz University of Medical Sciences, Tabriz, Iran; 3 Health Education and Health Promotion Department, School of Public Health, Tabriz University of Medical Sciences, Tabriz, Iran; University of South Alabama Mitchell Cancer Institute, UNITED STATES

## Abstract

**Aims:**

To evaluate the relationship between Mediterranean dietary pattern, anthropometric and metabolic biomarkers and vascular endothelial growth factor (VEGF) +405 G/C gene polymorphism in patient with metabolic syndrome (Mets).

**Materials and methods:**

In this study 150 patients with Mets and 50 healthy subjects were enrolled. Dietary intakes were evaluated with a semi-quantitative food-frequency questionnaire (FFQ) and Mediterranean dietary quality index (Med-DQI) was assessed. Anthropometric assessments and blood pressure measurement were performed. Biochemical assays including fasting serum glucose (FSG), matrix metalloproteinase-3 (MMP-3), liver enzymes and lipid profiles were also assessed. Polymorphism of +405 G/C VEGF gene was determined utilizing polymerase chain reaction-restriction fragments length polymorphism (PCR-RFLP) method.

**Results:**

Serum high density lipoprotein-cholesterol (HDL-C) was significantly lower and low density lipoprotein cholesterol (LDL-C), triglyceride (TG), total cholesterol (TC) concentrations and FSG were significantly higher in metabolic syndrome patients compared with control group (P < 0.05). Metabolic syndrome group with high consumption of “cholesterol” had significantly upper serum TG; also high consumption of “fish” and “vegetables-fruits” was associated with a significantly lower serum LDL concentrations. In metabolic syndrome patients with CC genotype, mean score of “saturated fatty acid” subgroup was significantly higher compared with other genotypes; whereas, in healthy individuals, mean score of “fruit-vegetable” subgroup in individuals of CC and GG genotype was significantly higher (P<0.05).

**Conclusion:**

Our findings indicated a significant relationship between Mediterranean dietary quality index and both anthropometric and metabolic risk factors. We also indicated a higher “saturated fatty acid” intake in CC genotype among metabolic syndrome patients.

## Introduction

Metabolic syndrome (Mets) is a growing public health problem worldwide; including a cluster of insulin resistance, dyslipidemia, central obesity, glucose intolerance, hypertension, alterations in several pro-inflammatory and inflammatory factors [1]; Moreover, Mets is associated with increased risk of type 2 diabetes mellitus and cardiovascular diseases [2]. Several studies have indicated that metabolic syndrome is associated with 3 to 4.3 fold increase in mortality from CVD and 3.5–5 increase in risk of diabetes mellitus (DM) [3]. The outbreak of metabolic syndrome in Iran is too high, pursuant to the Tehran Lipid and Glucose study (TLGS) in 2003, 42% of women and 24% of men were at risk of metabolic syndrome [4]. However, the current rampancy of metabolic syndrome may be higher up than the former assessment [5]. The National Cholesterol Education Program Adult Treatment Panel (ATP) III characterized metabolic syndrome as the presence of three or more of the following situations: triglyceride level of at least 150 mg/dl, HDL level less than 40 mg/dl in men and less than 50 mg/dl in women, systolic/diastolic blood pressure (BP) 130/85 mmHg or upper, fasting blood glucose (FBG) level 110 mg/dl or upper and waist circumference more than 102 cm in men and more than 88 cm in women [6]. The current explanations of central adiposity are based upon data from western populations; however, numerous studies reported that this cut-off probably requirements to be lowered for Asian populations. Several studied in Iran have reported that a more actual waist circumference for Iranians is > 90 cm for both men and women [7–9]. Therefore, the benefits of effective strategies to control the metabolic syndrome and an understanding of its components should be an important health concern [10].

Therapeutic approaches in metabolic syndrome are multi factorial, including exercise schedules, changing dietary habits and drug therapy [11]. In previous years a meta-analysis study on lifestyle modification and metabolic syndrome indicated that the proportion of people with resolved metabolic syndrome in the intervention group was approximately two fold than control group. The study also indicated that five out of six components of the syndrome decreased significantly in the intervention group compared with the control group [12]; Moreover, numerous reports demonstrated the beneficial role of diet and healthy food choices in prevention and treatment of metabolic syndrome [13].

Several studies have explored the efficacy of Mediterranean diet as one of the healthiest dietary patterns to protect chronic disease morbidity, higher life expectancy, and prevention of cardiovascular risks, Type 2 DM and non-alcoholic fatty liver [14]. This pattern is distinguished by a high intake of legumes, nuts, cereals, fruits and vegetables, a higher intake of olive oil with less saturated fats intake, a higher consumption of fish compared with meat and poultry intake, low to moderate dairy products intake and moderate alcohol intake regularly [15]. The beneficial roles of this dietary pattern are mainly attributed to its nutrient and non-nutrient compounds, including fibers, minerals, vitamins and phytochemicals [16]. Nearly all of the studies concentrated on determining the relations between Mediterranean dietary pattern and risk of disease; consequently, Mediterranean dietary quality index (Med-DQI), which developed by Gerber et al for the first time, is a beneficial tool to assess dietary quality emphasis on two various sources of fat (olive oil and saturated) and two various sources of protein (meat and fish) with reverse scores (Table 1) [17]. Considering the lack of knowledge about the relationship between Mediterranean dietary quality index and metabolic syndrome disease, the first hypothesis of the current study is evaluating the association between ingredients of Med-DQI with anthropometric and biochemical parameters of metabolic syndrome versus healthy group.

**Table 1:** Construction of the score for the Mediterranean Dietary Quality Index.

Scoring	0	1	2
**Saturated fatty acids (%energy)**	<10	10–13	>13
**Cholesterol (milligram)**	<300	300–400	>400
**Meats (gram)**	<25	25–125	>125
**Olive oil (milliliter)**	>15	15–5	<5
**Fish (gram)**	>60	60–30	<30
**Cereals (gram)**	>300	300–100	<100
**Vegetables+fruits (gram)**	>700	700–400	<400

Also, Previous studies have numerously reported the relationship between +405 G/C gene polymorphism of vascular endothelial growth factor (VEGF) (rs2010963) with essential hypertension and coronary artery diseases in diabetic patients [18, 19]; However, no study is available evaluating the gene-nutrient interaction of this polymorphism in patients with metabolic syndrome; therefore, second hypothesis of our study is evaluating the relationship between Med-DQI and VEGF gene polymorphism in patients with metabolic syndrome compared to healthy group according to various genotypes of VEGF +405 G/C gene polymorphisms.

## Materials and methods

### Design overview

The present case control study was carried out among 150 patients with metabolic syndrome and 50 healthy subjects. A detail description of the method is described previously [10]. Briefly, the study’s inclusion criteria included: having metabolic syndrome according to the National Cholesterol Education Program’s Adult Treatment Panel ш report (NCEP-ATP ш) criteria [20], (except for waist circumference which was characterized by 90 ≤ cm for both men and women in Iranian population [7, 8]), age 20 years or upper and living in Tehran; Moreover, exclusion criteria included: having a history of cardiovascular diseases, T2DM, cancer or renal diseases; being pregnant; taking medicine for hypertension or dislipidemia and having incomplete registration form. This study has been approved by the ethics committee of Tabriz University of Medical Science and Tehran University of Medical Science and written informed consent was obtained from all subjects before participation in the study.

### Anthropometric assessments

Weight was measured with a calibrated scale (SECA, Hamburg, Germany) to the nearest 0.1 kg while subjects were wearing light cloth and without shoes, height using a non-stretchable measurement tape with the precision of 0.1 cm. The body mass index (BMI) was calculated as weight (Kg) divided by height squared (m^2^) [21]. Waist circumference was measured in standing position at the level of the umbilicus, hip circumference (HC) was assessed at the maximum point between the hip and the buttock with a non-elastic tape.

### Biochemical assessments

After an overnight fasting, all of the subjects underwent a laboratory examination. Venous blood samples were taken from them and approximately 2 cc of the blood was transfused into tubes comprising ethylene diamine tetra acetic acid (EDTA) for genetic assays; moreover, sera were extracted from residual blood samples for biochemical assays including fasting serum glucose (FSG), alanine aminotransferase [22], aspartate aminotransferase [23], total cholesterol (TC), triglyceride (TG), high-density lipoprotein cholesterol (HDL-C), adiponectin and matrix metalloproteinase-3(MMP3). Serum AST, ALT, TC, FSG, TG and HDL-C were analyzed by enzymatic colorimetric method (Pars Azmoon, Tehran-Iran), Serum LDL-C was calculated by Friedewald formula [24]. Serum matrix metalloproteinase-3 was also analyzed by ELISA method (Hangzhou Eastbiopharm Co, USA) with sensitivity of 0.01ng/ml and assay range of 0.05–10 ng/ml.

### Dietary intake

Usual dietary intake was evaluated using a 147 item semi-quantitative food-frequency questionnaire (FFQ) which was validated for use in Iranian society [25]. The FFQ consisted of a specified list of standard serving sizes consumed by Iranians popularly. Subjects reported their frequency intake of a given food item during the previous year on a daily, weekly or monthly basis. The foods consumption and portion sizes for every food items were transformed to a daily intake. Then, we calculated the diet score on the basis of Mediterranean diet quality index (Med-DQI) (Table 1). The index allocates a score of 0, 1 or 2 according to the seven components intake and then final score was reported as a summation of all nutrient scores ranged between 0 and 14. A lower score of index denotes better diet quality [17].

### Determination of the VEGF +405 C/G polymorphism genotype

**Fig 1:**
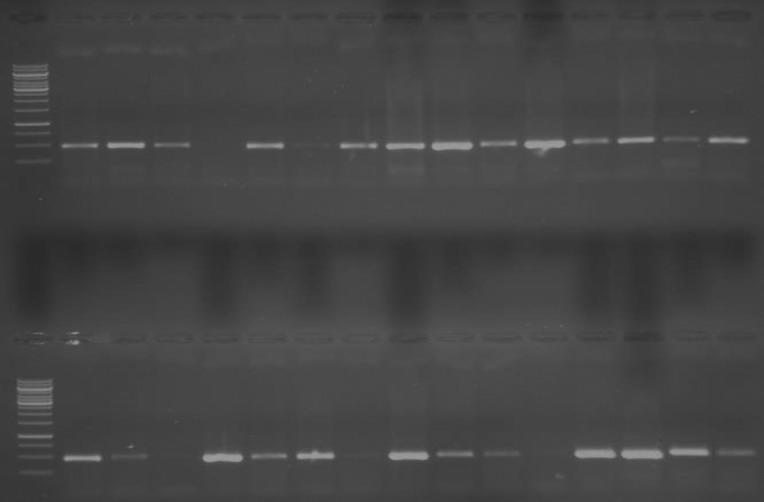
PCR–RFLP analysis for +405 VEGF gene polymorphism.

High molecular weight genomic DNA was isolated from peripheral blood leukocytes by the standard procedures [18]. Polymerase chain reaction- restriction fragment length polymorphism (PCR-RFLP) was employed to determine the +405 VEGF C/G (rs2010963) polymorphism status with technical specifications (Fig 1). PCR was performed in a volume of 10 μL including 100 ng DNA, 25 μL Taq PCR master mix RED, 2.5 μL primers and 12.5 μL deionized water. Genomic DNA was amplified in a final volume of 10 μl using the following conditions: denaturation at 95°C for 5 min followed by 35 cycles at 94°C for 1 min, 60°C for 1.5 min (annealing) and 72°C for 2 min (extension). A final extension was at 72°C for 10 min. For the VEGF +405 polymorphism the PCR product was digested with the *Bsm*FI restriction nuclease (New England Biolabs, USA). The amplification products were separated by electrophoresis through 1% agarose gel stained with ethidium bromide. For the VEGF +405 polymorphism the uncut fragment was 469 bp (C allele) and digestion products were 195 bp and 274 bp (G allele).

### Statistical analysis

Statistical analyses were performed by SPSS software version 16 (SPSS Inc., Chicago, IL, USA). Kolmogorov–Smirnov test was performed for normality of the distributions of variables. The comparison of discrete and continuous variables between groups was performed by Chi- square test and independents sample t test or one-way ANOVA tests, respectively. The comparison of anthropometric or biochemical variables between different categorizes of Med-DQI pattern, was performed by ANCOVA after adjusting for the confounder effects of age and gender. P values less than 0.05 was considered as statistically significant.

## Results

As described earlier, metabolic syndrome, as a cluster of dyslipidemia, central obesity, glucose intolerance and hypertension, is a growing public health problem and attracted a much attention worldwide; in the results section, we are reporting our findings according to ingredients of the subsections of metabolic syndrome ingredients as follows:

### Metabolic syndrome and anthropometric parameters

Since metabolic syndrome is associated with obesity, we decided to examine the relationship between metabolic syndrome and obesity related factors. We employed the anthropometric parameters in of study participants. As shown in Table 2, WC, WHR and BMI were significantly higher in metabolic syndrome group compared to healthy group (P < 0.05).

**Table 2:** General demographic, anthropometric and biochemical parameters in patients with metabolic syndrome compared to healthy controls.

Variable	Patients with Metabolic Syndrome (N = 150)	Healthy control (N = 50)	P value
**Age (y)**	44.01 ± 10.08	37.97±8.80	**0.001**
**Gender**	66.5%	33.5%	**0.001**
**Weight (kg)**	87.47 ± 14.91	85.21 ± 12.24	**0.001**
**Height (cm)**	169.95 ± 9,85	163.48 ± 10.21	0.33
**BMI (kg/m**^**2**^**)**	30.27 4.68	31.89 3.89	**0.03**
**WC (cm)**	104.40 ± 8.69	92.89 ± 8.03	**0.004**
**HC (cm)**	111.94 ± 9.76	112.33 ± 8.39	0.81
**WHR**	0.93 ± 0.07	0.89 ± 0.06	**0.001**
**FSG (mg/dl)**	108.84 ± 13.79	90.43 ± 8.01	**0.05**
**TC (mg/dl)**	194.66 ± 39.49	190.02 ± 25.57	**0.03**
**TG (mg/dl)**	190.61± 112.50	136.61 ± 60.29	**<0.0001**
**LDL (mg/dl)**	128.25 ± 33.00	113.60± 22.92	**0.002**
**HDL (mg/dl)**	41.44 ± 9,95	48.91 ±11.44	**0.001**
**AST (IU/l)**	19.82± 10.29	23.91 ± 11.10	0.45
**ALT (IU/l)**	21.89 ± 10.35	23.33 ± 5.50	0.35
**MMP-3 (ng/ml)**	4.27 ± 3.32	3.12 ± 2.35	0.56

BMI, body mass index; WC, waist circumference; HC, hip circumference; WHR, waist to hip ratio; FSG, fasting serum glucose; TC, total cholesterol; TG, triglyceride; LDL, low density lipoprotein cholesterol; HDL, high density lipoprotein cholesterol; AST, aspartate aminotransferase; ALT, alanine aminotransferase; MMP-3, matrix metalloproteinase-3. P value for gender based on Chi-Square Test; otherwise based on independent T-test using equal variable. Continuous variables data are presented based on mean (SD).

### Metabolic syndrome, lipid profile and FSG

An association between dyslipidemia, glucose intolerance and metabolic syndrome has been documented. Since these parameters may underlie the constellation of risk factors involved in the development of type 2 diabetes and cardiovascular diseases; therefore, the concentration of related biochemical parameters were assayed. As shown in Table 2, LDL-C, TG and TC concentrations were significantly higher and serum HDL-C was significantly lower in metabolic syndrome group compared to control group (P < 0.05).

Moreover, FSG concentration was significantly higher in metabolic syndrome group. As expected, higher lipid concentrations will be a potent risk factor of cardiovascular events in patients with metabolic syndrome.

Moreover, one of the beneficial approaches in the treatment and prevention of metabolic syndrome is healthy dietary patterns and healthy dietary choices. Also, the relationship between Med-DQI and +405 G/C gene polymorphism, as a novel approach of gene-nutrient interaction has not been previously reported. We determined to explore the preventive efficacy of Med-DQI in patients with metabolic syndrome versus control group.

### Med-DQI and anthropometric parameters

Comparison of anthropometric variables in different categorizes of Med-DQI components in patients with metabolic syndrome and in healthy group are reported in Tables 3 and 4, respectively. Patients with metabolic syndrome with upper scores of “cholesterol” and “olive oil” subgroups had significantly higher WC; also, patients with upper score of “fish” had significantly higher WC and BMI among anthropometric parameters; consequently, in healthy control group upper score of “cereal” was associated with significantly higher BMI and WHR. Upper score of “saturated fatty acid” and “cholesterol” subgroups were also associated with higher WC and BMI in healthy control group, respectively (Tables 3 and 4).

**Table 3:** Comparison of anthropometric parameters and blood pressure according to components of Med-DQI in patients with metabolic syndrome.

Characteristics	Metabolic syndrome(N = 150)					
Saturated fatty acid (n)	BMI	HC	WC	WHR	SBP	DBP
**0(121)**	30.04 ±4.63	112.39±10.28	104.05±8.76	0.92±0.06	132.19±11.74	88.25±6.57
**1(25)**	30.55±5.24	109.70±7.57	104.00±8.04	0.94±0.10	130.00±8.53	89.20±5.89
**2(6)**	29.78±4.09	108.83±12.40	106.00±9.40	0.98±0.12	133.33±8.16	85.83±3.76
**P**	0.87	0.46	0.86	0.14	0.63	0.50
**Cholesterol (n)**						
**0 (106)**	29.82±4.34	111.56±9.30	103.04±8.29	0.92±0.07	131.48±10.83	88.13±5.53
**1 (21)**	31.43±4.70	113.14±15.08	108.09±7.07	0.94±0.08	130.00±8.21	88.80±6.69
**2(23)**	30.25±6.10	111.52±8.52	106.56±10.36	0.95±0.05	135.43±14.21	88.69±9.44
**P**	0.35	0.86	**0.03**	0.25	0.21	0.86
**Meats (n)**						
**0(39)**	29.01±4.15	111.23±8.18	102.65±7.81	0.92±0.06	130.00±7.16	87.56±3.60
**1(106)**	30.46±4.72	111.94±10.45	104.64±8.83	0.93±0.08	132.47±12.23	88.70±6.86
**2 (7)**	30.81±6.84	111.83±13.81	104.85±10.57	0.96±0.06	129.28±6.07	87.85±10.35
**P**	0.24	0.95	0.46	0.52	0.40	0.62
**Olive oil (n)**						
**0 (2)**	34.97±1.82	119.50±7.77	104.57±10.19	0.98±0.02	135.00±7.07	95.00±7.07
**1 (28)**	30.97±5.86	110.87±8.52	103.80±8.14	0.92±0.06	132.32±10.49	89.28±7.54
**2(122)**	29.83±4.37	111.75±10.30	117.50±4.94	0.93±0.07	131.72±11.38	87.98±6.05
**P**	0.17	0.52	**0.04**	0.53	0.89	0.20
**Fish (n)**						
**0(2)**	30.17±2.52	105.50±8.34	115±9.89	0.91	130.00	90.00±14.14
**1(4)**	29.97±4.63	111.80±9.90	104.25±3.94	0.99±0.09	131.25±6.29	87.50±2.88
**2 (144)**	39.50±5.65	133.00±8.12	103.98±8.67	0.93±0.07	131.72±11.12	88.37±6.36
**P**	**0.01**	**0.04**	0.20	0.28	0.97	0.90
**Cereal (n)**						
**0(148)**	30.14±4.71	111.78 ±10.0	104.14±8.65	0.93±0.07	131.90±11.21	88.32±6.41
**1(2)**	27.90±0.69	110.00	102.50± 7.77	0.88	130.00	87.50 ± 3.53
**2(0)**		-	-		-	
**P**	0.50	0.86	0.79	0.50	0.81	0.85
**Fruits and vegetables (n)**						
**0(0)**	31.50±6.80	111.50±5.99	105.12±9.70	0.94±0.06	133.75±16.85	90.00±9.25
**1(12)**	29.92± 4.69	111.85±7.49	104.60 ±8.98	0.92±0.05	132.54± 7.89	87.84±6.72
**2(38)**						
	30.17± 4.42	111.80±11.37	104.23±8.50	0.93±0.09	131.25±11.96	88.34±6.19
**P**	0.67	0.99	0.94	0.53	0.69	0.67

BMI, body mass index; WC, waist circumference; HC, hip circumference; WHR, waist to hip ratio; SBP, systolic blood pressure; DBP, diastolic blood pressure.

**Table 4:** Comparison of anthropometric parameters according to components of Med-DQI in healthy control group.

Characteristics	Healthy controls (N = 50)					
Saturated fatty acid (n)	BMI	HC	WC	WHR	SBP	DBP
**0(30)**	30.84 ±4.26	112.50±8.67	98.31±8.13	0.90±0.06	118.68±13.35	80.32±7.32
**1(13)**	31.80±4.14	109.63±7.97	101.24±6.11	0.88±0.08	120.32±15.41	85.34±7.86
**2(7)**	34.26±3.56	119.10±2.92	109.00±7.35	0.91±0.09	117.51±15.75	88.39±8.24
**P**	0.39	0.24	**0.04**	0.74	0.31	0.81
**Cholesterol (n)**						
**0 (32)**	30.80±3.90	113.09±8.52	100.10±7.11	0.90±0.07	117.32±13.51	87.24±8.36
**1 (12)**	29.95±4.17	112.08±7.79	100.12±10.69	0.89±0.07	118.21±12.61	87.95±8.97
**2(6)**	34.06±4.30	103.10±5.93	96.20±13.29	0.90±0.04	118.74±12.75	82.67±7.92
**P**	**0.03**	0.27	0.80	0.93	0.37	0.61
**Meats (n)**						
**0(13)**	31.14±4.04	113.34±10.65	100.51±9.61	0.87±0.07	131.65±14.40	81.94±6.33
**1(37)**	31.40±4.29	112.10±7.97	99.75±7.79	0.90±0.07	121.35±15.01	83.25±8.68
**2 (0)**	-	-	-	-	-	-
**P**	0.88	0.73	0.82	0.44	0.80	0.16
**Olive oil (n)**						
**0 (5)**	31.3	108.50	95.40	0.90	101.63±14.98	73.98±8.75
**1 (6)**	28.30±4.66	104.05±7.28	93.40±9.33	0.94±0.09	117.25±11.23	82.29±7.58
**2(39)**	31.53±4.23	112.93±8.38	100.40±8.01	0.89±0.07	121.57±15.21	88.96±7.38
**P**	0.58	0.32	0.42	0.60	0.51	0.76
**Fish (n)**						
**0(7)**	31.20	107.00	98.90	0.82	131.26±15.74	91.86±7.37
**1(0)**	-	-	-	-	-	-
**2 (43)**	31.35±4.25	112.48±8.46	99.92±8.14	0.90±0.07	12.00±14.32	89.37±6.72
**P**	0.97	0.52	0.90	0.29	0.49	0.36
**Cereals (n)**						
**0(42)**	30.92±3.79	112.28±8.36	100.22±8.08	0.89±0.06	141.36±14.63	76.34±9.41
**1(8)**	38.80±5.09	113.25±12.37	94.05± 5.02	0.99±0.07	133.87±13.02	78.64±8.94
**2(0)**		-	-		-	
**P**	**0.008**	0.87	0.29	**0.05**	0.39	0.51
**Fruits and vegetables(n)**						
**0(0)**	-	-	-	-	-	-
**1(11)**	31.44± 3.75	110.82±7.39	98.80 ±4.88	0.92±0.07	132.53±14.36	89.16±6.30
**2(39)**	31.34± 4.31	112.57±8.62	100.06±8.46	0.89±0.07	153.28±15.02	98.56±8.13
**P**	0.96	0.67	0.74	0.45	0.41	0.78

BMI, body mass index; WC, waist circumference; HC, hip circumference; WHR, waist to hip ratio; SBP, systolic blood pressure; DBP, diastolic blood pressure

As previously mentioned, the upper scores of Med-DQI ingredients, denotes the worse dietary quality; therefore, we can conclude that the patients with lower fish, cereals and higher saturated fatty acid and dietary cholesterol intake are more obese and therefore at higher risk of obesity-related comorbidities.

### Med-DQI and biochemical parameters

Among biochemical variables, metabolic syndrome patients with upper scores of “fish” and “vegetable and fruit” subgroups had significantly higher LDL and patients with upper score of “cholesterol” had higher serum TG concentrations; consequently, upper score of “vegetable and fruit” was associated with higher TC and LDL in healthy group (Tables 5 and 6). In other word, lower dietary intake of vegetables and fruits, fish and high intake of cholesterol had higher risk of dyslipidemia.

**Table 5:** Comparison of biochemical parameters according to components of Med-DQI in patients with metabolic syndrome.

Characteristics	Metabolic syndrome (N = 150)						
	FSG(mg/dl)	TG(mg/dl)	TC(mg/dl)	ALT(IU/l)	AST(IU/l)	HDL(mg/dl)	LDL(mg/dl)
**Saturated fatty acid (n)**							
**0 (119)**	89.98±13.83	190.52±108.97	199.28±40.13	20.23±10.31	22.23±10.44	41.80±10.19	131.44±33.76
**1 (25)**	90.16±15.50	182.20±135.75	181.08±36.67	16.75±6.06	19.16±6.27	42.16±9.46	116.56±25.02
**2 (6)**	88.83±14.90	190.50±75.51	190.66±33.51	9.50±0.70	15.00±7.07	37.66±7.86	125.83±24.26
**P**	0.97	0.94	0.10	0.19	0.40	0.59	0.11
**Cholesterol (n)**							
**0 (106)**	89.76±13.13	170.13±69.86	197.34±42.76	19.20±9.54	20.56±8.97	41.55±10.20	129.80±34.03
**1 (21)**	94.95±17.53	178.09±74.53	194.19±29.65	20.18±10.47	25.27±10.85	41.28±9.05	129.38±30.19
**2 (23)**	86.34±14.14	194.28±124.89	191.00±33.07	18.00±11.89	22.00±15.03	42.73±10.04	123.39±27.36
**P**	0.12	**0.04**	0.76	0.91	0.35	0.85	0.69
**Meats (n)**							
**0 (39)**	89.87± 12.43	183.58 ± 117.61	201.71±48.91	37.63± 21.99	25.25± 9.21	41.51 ±9.07	130.74±41.49
**1 (104)**	90.00± 15.05	191.13 ±113.78	192.53±35.87	51.21± 26.28	33.83± 15.29	41.80± 10.38	127.34±28.23
**2 (7)**	88.42±6.92	196.00±60.31	213.14±38.09	66.00	39.00	41.85±10.35	137.57±40.88
**P**	0.96	0.92	0.23	0.83	0.94	0.98	0.65
**Olive oil (n)**							
**0 (2)**	93.50±2.12	132.50±54.44	169.00±12.72	9.00	10.00	39.50±4.94	119.50±4.94
**1 (28)**	89.92±15.91	192.89±98.99	201.85±35.68	19.80±10.68	17.10 ±8.68	43.42±9.87	133.14±30.15
**2 (120)**	89.91±13.78	189.22±115.81	195.03±40.77	19.36±9.63	22.45 ±9.78	41.33±10.07	127.92±33.28
**P**	0.93	0.76	0.45	0.57	0.14	0.58	0.68
**Fish (n)**							
**0 (2)**	88.50±10.60	225.00±39.59	204.00±53.74	19.50±8.70	19.43±7.02	55.00±4.24	118.00±69.29
**1 (4)**	93.25±8.53	155.00±75.21	214.75±41.93	19.66±9.50	20.00±12.49	41.00±5.29	153.25±42.43
**2 (144)**	89.82±14.28	189.86±113.95	195.22±39.81	19.25± 9.90	21.52 ±.9.76	41.57 ±10.4	158.16±31.88
**P**	0.88	0.75	0.60	0.94	0.79	0.16	**0.02**
**Cereal (n)**							
**0 (148)**	90.01±14.09	189.81±112.68	195.92±39.97	19.51± 9.77	21.50± 9.93	41.66±10.03	129.12±32.41
**1 (2)**	86.50±14.84	139.50±17.67	198.00±12.72	11.50±0.70	20.00±2.82	44.00±5.65	102.50±37.47
**2 (0)**	-	-	-	-	-	-	-
**P**	0.72	0.53	0.94	0.25	0.83	0.74	0.25
**Fruits and vegetables (n)**							
**0(8)**	91.12 ±5.51	230.62±108.84	183.37±29.80	12.66± 11.71	12.66±8.32	41.75±14.32	107.00±23.13
**1(51)**	90.62 ±14.93	193.92±84.58	203.49±36.13	20.14± 11.22	24.04 ±11.16	42.07±10.19	133.33±30.98
**2(91)**	89.44± 13.68	186.15±124.43	191.62±41.50	19.93±9.82	21.37± 9.80	41.03±9.50	131.99±33.02
**P**	0.85	0.54	0.15	0.48	0.18	0.82	**0.05**

Fasting serum glucose; TG, triglyceride; TC, total cholesterol; LDL, low density lipoprotein cholesterol; HDL, high density lipoprotein cholesterol; AST, aspartate aminotransferase; ALT, alanine aminotransferase; MMP-3, matrix metalloproteinase-3.

**Table 6:** Comparison of biochemical parameters according to components of Med-DQI in healthy control group.

Characteristics	Healthy controls (N = 50)						
	FSG(mg/dl)	TG(mg/dl)	TC(mg/dl)	ALT(IU/l)	AST(IU/l)	HDL(mg/dl)	LDL(mg/dl)
**Saturated fatty acids (n)**							
**0(29)**	90.68 ±7.85	136.44±59.67	189.88±29.03	27.88±11.96	23.16±5.85	49.56±11.40	112.30±25.82
**1(13)**	90.77±8.67	119.00±67.69	186.22±23.08	28.55±10.92	22.66±5.00	50.11±12.60	112.31±16.04
**2(8)**	87.33±10.01	172.00±32.44	202.66±12.50	26.33±4.04	26.66±3.21	40.00±5.56	128.26±8.80
**P**	0.79	0.41	0.66	0.95	0.54	0.37	0.52
**Cholesterol (n)**							
**0 (31)**	90.55±8.23	146.62±61.53	190.11±28.86	28.51±11.82	23.51±5.99	47.55±11.97	112.92±24.57
**1 (12)**	92.12±7.33	114.37±49.48	195.25±19.06	25.50±8.22	22.37±3.85	53.75±8.89	118.62±19.81
**2(7)**	82.00±2.82	87.00±60.81	168.00±4.24	29.50±16.26	41.50±6.36	48.00±14.14	102.60±2.26
**P**	0.28	0.20	0.44	0.78	0.84	0.41	0.66
**Meats (n)**							
**0(13)**	89.28±6.01	142.42±56.75	204.57±20.47	25.28±9.01	22.85±4.05	51.28±11.64	124.80±12.62
**1(37)**	90.70±8.48	135.03±61.93	186.63±26.95	28.53±11.58	23.43±5.84	48.36±11.53	110.98±24.12
**2 (0)**	-	-	-	-	-	-	-
**P**	0.68	0.77	0.10	0.49	0.80	0.55	0.15
**Olive oil (n)**							
**0 (5)**	95.00	115.00	193.00	38.00	28	43.00	118.80
**1 (6)**	84.00	83.50±55.86	185.50±20.50	18.50±0.70	19.50 ±0.70	59.00±1.41	109.80± 7.91
**2(39)**	90.00±8.18	140.17±60.60	190.20±27.49	28.17±11.22	23.41± 5.60	48.50±11.64	113.67± 23.86
**P**	0.45	0.41	0.96	0.32	0.44	0.40	0.95
**Fish (n)**							
**0(8)**	94.00	67.00	188.00	12.00	20.00	59.00	115.60
**1(0)**	-	-	-	-	-	-	-
**2 (42)**	90.33±8.10	138.36±59.98	190.08±26.94	28.36±10.92	23.41±5.51	48.63±11.48	113.54±23.24
**P**	0.65	0.24	0.94	0.14	0.54	0.37	0.93
**Cereal (n)**							
**0(42)**	90.45±8.24	138.11 ±60.71	190.62± 27.15	27.71± 11.30	22.94± 5.36	48.51±11.60	114.25 ±23.40
**1(8)**	90.00± 1.41	107.00±60.81	179.50± 10.60	31.50± 7.7	28.00± 4.24	56.00 ± 5.65	102.10± 4.10
**2(0)**	-	-	-	-	-	-	-
**P**	0.93	0.48	0.57	0.64	0.12	0.37	0.47
**Fruits and vegetables (n)**							
**0(0)**	-	-	-	-	-	-	-
**1(12)**	93.20± 8.52	148.00±57.70	186.84 ±24.29	28.40± 12.75	24.80± 5.35	50.80±9.36	111.03± 20.40
**2(38)**	90.00± 7.99	134.62±61.37	210.40±34.35	27.84± 11.04	23.09 ±5.57	48.62±11.84	130.00± 33.35
**P**	0.41	0.65	**0.02**	0.91	0.52	0.69	**0.04**

Fasting serum glucose; TG, triglyceride; TC, total cholesterol; LDL, low density lipoprotein cholesterol; HDL, high density lipoprotein cholesterol; AST, aspartate aminotransferase; ALT, alanine aminotransferase; MMP-3, matrix metalloproteinase-3.

### Med-DQI and +405 G/C gene polymorphism

Most recently, gene nutrient interactions has attracted a great attention because of the important roles of genes and different genotypes in the individual response to environmental parameters like diet and dietary practices.

As we showed in the comparison of components of Med-DQI according to +405C/G of VEGF gene polymorphism between study groups, the score of “saturated fatty acid” subgroup in metabolic syndrome patients with CC genotype was higher compared with patients in other genotypes; Moreover, the score of “vegetable and fruit” subgroup in healthy control group with CC and GG genotypes was higher than GC genotype of VEGF gene (Table 7).

**Table 7:** Comparison of components of Med-DQI according to +405 G/C of VEGFgene polymorphism between study groups.

Characteristics	Metabolic syndrome (N = 150)			P	Healthy controls (N = 50)			P
*Genotype*	*GG*	*GC*	*CC*		*GG*	*GC*	*CC*	
**Saturated fatty acids**	0.052±0.22	0.28±0.57	0.41±0.51	**0.01**	0.36±0.67	0.36±0.59	0.57±0.78	0.76
**Cholesterol**	0.31±0.74	0.44±0.79	0.50±0.79	0.77	0.09±0.30	0.36±0.49	0.57±0.97	0.20
**Meats**	0.89±0.45	0.79±0.53	0.66±0.49	0.48	0.72±0.46	0.84±0.37	0.85±0.37	0.71
**Olive**	1.89±0.31	1.79±0.40	1.75 ±0.45	0.54	2.00±0.00	1.84±0.50	1.85±0.37	0.56
**Fish**	1.94±0.22	1.93±0.31	2.00±0.00	0.78	2.00±0.00	1.89±0.45	2.00±0.00	0.63
**Cereals**	-	0.020±0.14	-	0.73	-	0.10±0.31	-	0.38
**Fruits and vegetables**	1.45±0.73	1.64±0.56	1.67±0.095	0.36	2.00±0.00	1.73±0.45	2.00±0.00	**0.03**

In other word, patients with CC or GG genotypes of +405C/G of VEGF gene polymorphism are at greater risk of metabolic syndrome health consequences and its related disturbances like diabetes or CVD.

## Discussion

In the present study, according to the components of Med-DQI, patients with high scores of dietary cholesterol and olive oil had significantly higher WC. Moreover, we observed significantly upper serum LDL and TG concentrations in patients with higher scores of fish, vegetables-fruits and cholesterol, respectively. Studies regarding the direct effect of Mediterranean dietary pattern on metabolic syndrome are rarely; however, several studies reported the protective role of dietary pattern against known risk factors of metabolic syndrome such as obesity [26], insulin resistance [27], hypertension and dyslipidemia [28]. In fact, over the past decades, epidemiological studies have been suggested that nutritional factors has the most important effects against biochemical and anthropometric abnormalities in metabolic syndrome which among them dietary pattern close to the Mediterranean diet demonstrated the most beneficial role [28]. In a sub-analysis of the EPIC study involving a cohort of 497308 subjects, a high adherence to the Mediterranean diet was associated with significantly lower values of anthropometric parameters including BMI and WC during three years [29]. In the SUN cohort study analyzing 9408 men and women during six years, high adherence to the Mediterranean diet was associated with prevention of age-related changes in blood pressure [30]. Moreover, in the ATTICA study, adherence to the Mediterranean dietary pattern was contributed to 11.4% better insulin sensitivity, 13% lower concentrations of serum total cholesterol and 3 mmHg lower values of systolic blood pressure in overweight and obese subjects [31]. In the study by Williams et al, higher adherence to a dietary pattern similar to Mediterranean diet with high content of fruits and vegetables and high monounsaturated fatty acids was negatively associated to features of metabolic syndrome [32].

The Med-DQI is a beneficial tool for dietary quality assessment and has been validated previously using nutritional biomarkers [17]. This index was based upon the commendations regarding the diet and health of National Research Council and American Heart Association [23]. These commendations are included of intake of less than 30% total energy from fat per day, less than 10% of total energy from saturated fat, less than 30 mg from cholesterol, 55% of energy from complex carbohydrates and more than 5 servings of fruits and vegetables.

According to the scores of Med-DQI, we represented upper serum TG concentrations in patients with metabolic syndrome with high score of cholesterol (P<0.05). Serum triglyceride, rapidly responses to a fatty meal in a dose-dependent manner and its concentrations in serum increases proportional to the dietary fat amount [33]. It has also been reported that baseline serum triglyceride is a vital determinant of its response to a high fat diet; actually, a low-fat diet is associated with lower serum triglyceride concentrations in patients with hard hyper-triglyceridemia [34].

We also observed the beneficial roles of fish and fruit-vegetable components in reducing serum LDL concentrations in patients with metabolic syndrome. The advantageous effects of fish and fruit-vegetable on obesity [21], dislipidemia [35] and insulin resistance [36] had been reported in past [37]. There is a possible mechanism by which dietary fish oil may exert their hypolipidemic action that is reduction of VLDL synthesis in the liver. Fatty acid synthesis in rat liver is reduced by the omega-3 fatty acids of fish oil, which may result in inhibited VLDL synthesis. It is proposed that a relative block in the conversion of VLDL to LDL was occurred, thus allowing a reduction of LDL [38]. Moreover, the effects of vegetables and fruits on LDL levels contribute to their role in reducing hepatic cellular cholesterol concentration that cause to up-regulation of the LDL receptor, increased hepatic cholesterol uptake and decline in serum levels as observed; also, diet rich in fruits and vegetables are a source of dietary fiber [39]; In this regard, a meta-analysis study indicated LDL reducing effects of dietary fiber [22].

In the current study, higher score of “saturated fatty acid” in CC genotype of VEGF gene polymorphisms in patients with metabolic syndrome was also reported. Previous studies indicated a key role of VEGF in response against oxidative stress in metabolic syndrome, dislipidemia and insulin resistance. VEGF exerts its effects mostly by its antigenic roles that cause to increase permeability and widening of blood vessels [40]; therefore, VEGF may be a therapeutic target for management of oxidative stress in metabolic disorders. The relationship of +405 G/C gene polymorphism of VEGF with essential hypertension and coronary artery diseases in diabetic patients has been reported previously [18, 19]; However, considering the lack of knowledge about the relationship between Mediterranean dietary quality index and VEGF gene polymorphism in patients with metabolic syndrome, further interventional studies are needed to evaluate Mediterranean dietary quality index in patients with metabolic syndrome disease according to various genotypes of VEGF +405 G/C gene polymorphisms.

## Conclusion

The present study represented a significant relationship between components of Mediterranean dietary pattern and both anthropometric and metabolic risk factors in patients with metabolic syndrome. We also represented a gene-nutrient interaction between CC genotype of VEGF +405 G/Cgene polymorphisms and dietary saturated fatty acid intakes in metabolic syndrome. It is obvious that dietary modification is the most efficient way to decline chronic disease risk factors [41]. This is the first study evaluating the effect of Mediterranean dietary pattern on anthropometric and metabolic parameters of metabolic syndrome considering the interactive role of VEGF gene polymorphism; although several limitations of the present study should also be addressed: firstly the case control design of the study has not potential to address cause-effect relationship between variables. Moreover, we did not assess the serum concentrations of VEGF in the current study due to financial limitations. More studies with interventional designs are needed to better evaluate the effect of Mediterranean dietary pattern on risk factors of metabolic syndrome and the VEGF gene expression.

## Supporting information

S1 Dataset Dataset. (SAV)

## Abbreviations

BMI: body mass index
LDL: low density lipoprotein concentrations
VLDL: very low density lipoprotein concentrations
HDL: high density lipoprotein concentrations
VEGF: vascular endothelial growth factor
WHO: World Health Organization
TC: total cholesterol
(NCEP-ATP III): National Cholesterol Education Program’s Adult Treatment Panel III report
